# Regulating the Plasticity of Hippocampal Neurons via Electroacupuncture in Depression Model Mice

**DOI:** 10.1111/cpr.70057

**Published:** 2025-05-15

**Authors:** Yiyang Wang, Xinyi Du, Chenxi Duan, Miaomiao Wang, Ying Zhu, Lihua Wang, Jun Hu, Yanhong Sun

**Affiliations:** ^1^ Shanghai Institute of Applied Physics Chinese Academy of Sciences Shanghai China; ^2^ University of Chinese Academy of Sciences Beijing China; ^3^ Institute of Materiobiology, College of Sciences Shanghai University Shanghai China

**Keywords:** depression, electroacupuncture, hippocampus, neuronal plasticity

## Abstract

Effective treatment of depression poses a major clinical challenge, accompanied by considerable social and emotional burdens. Electroacupuncture, a non‐pharmacological modality derived from traditional Chinese medicine, offers a promising alternative for depression treatment due to its safety and efficacy. However, its underlying molecular mechanisms remain unclear. In this study, a corticosterone‐induced depression model in C57BL/6 mice was employed and electroacupuncture was applied to stimulate at Zusanli (ST36) acupoint. The results demonstrated that electroacupuncture effectively alleviated depression‐like symptoms and restored the structural morphology and plasticity of neurons in the hippocampal CA1 region. Further analysis revealed a significant upregulation of brain‐derived neurotrophic factor (BDNF) and β‐type calmodulin‐dependent protein kinase II (CaMKIIβ), which are associated with neuronal plasticity regulatory pathways. This study elucidates the potential molecular mechanisms by which electroacupuncture alleviates depression through the regulation of neuroplasticity, providing an experimental basis for its clinical application.

## Graphical abstract

This study explores the regulation of the plasticity of hippocampal neurons via electroacupuncture in corticosterone‐induced depression model mice. Electroacupuncture at the Zusanli (ST36) acupoint alleviated depression‐like symptoms and enhanced neuronal plasticity in the hippocampal CA1 region by upregulating BDNF and CaMKIIβ, highlighting its underlying mechanism in depression treatment.

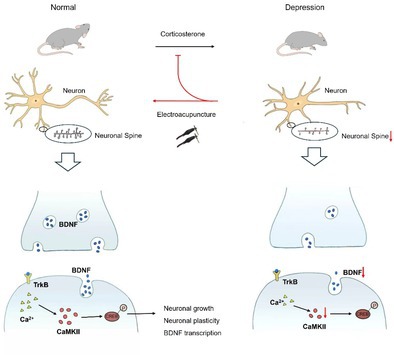

## 1 Introduction

Depression is one of the most prevalent mental disorders worldwide, triggered by various factors such as chronic stress stimulation or physiological causes. It is characterised by persistent depressive moods that are difficult to self‐regulate. In severe cases, it affects social skills and autonomous behaviour and may even lead to suicidal behaviour. The onset of depression is often accompanied by pathological changes in the brain, including hippocampal atrophy, astrocytic degeneration, reduced neuronal integrity and a decrease in dendritic spine density [1, 2, 3]. As a key brain region related to depression, the abnormal function of the hippocampus is closely related to the onset and development of depressive symptoms. Reduced hippocampal volume is one of the markers of major depression, elevating the risk of depression by four to eight times [4]. Among the structural changes in the hippocampus associated with depression, the CA1 region exhibits significant volume reduction, which correlates with the duration of the disease. In addition, the functional connectivity of neurons is markedly diminished [5, 6]. A reduction in CA1 volume leads to an imbalance in the tryptophan (Trp) metabolic pathway and overexpression of β‐type calmodulin‐dependent protein kinase II (CaMKIIβ), which activates depression‐associated neuroinflammatory responses [7, 8].

The decline of hippocampal synaptic plasticity is an important pathological change in depression. Neuronal plasticity includes synaptic plasticity and neurogenesis, and the alteration of these processes can lead to dysfunction in emotional regulation. The regulation of neuronal plasticity involves a series of complex biochemical molecules and regulatory pathways. Brain‐derived neurotrophic factor (BDNF) is a crucial neurotrophic factor and plays an important role in the growth and development of brain neurons. BDNF binds to the receptor tyrosine kinase B (TrkB), activating the BDNF/TrkB pathway and promoting the phosphorylation of cAMP‐response element binding protein (CREB) [9, 10]. In addition, the BDNF/TrkB pathway activates phospholipase C‐gamma (PLC‐γ) signalling, inducing calcium ions binding. This process phosphorylates CaMKII, generating p‐CaMKII, which in turn activates CREB. As a transcription factor, CREB regulates the transcription of BDNF and toll‐like receptor 1 (TLR1) associated with neuroinflammation [11, 12, 13]. By enhancing the plasticity of hippocampal neurons, promoting the formation of synaptic connections and neuronal regeneration, it may provide new strategies for the treatment of depression.

Current clinical treatments for depression include drug therapy, physical therapy, and psychological intervention. Although the symptoms and quality of life of patients have been improved to a certain extent, some patients find it difficult to achieve the ideal effect. In recent years, acupuncture has attracted attention in the treatment of depression. Electroacupuncture (EA), as a kind of physical therapy in traditional medicine, regulates the physiological and psychological state of the human body by stimulating specific acupoints. It offers significant advantages in treating depression, as it involves physical stimulation without concerns about drug transport across the blood–brain barrier or potential systemic toxic side effects. Other physical therapies such as electroconvulsive therapy (ECT), repetitive transcranial magnetic stimulation (rTMS), transcranial direct current stimulation (tDCS) and deep brain stimulation (DBS) are commonly employed for major depression [14]. Some of these treatments require sophisticated surgical implantation of electrodes or are performed under anaesthesia, with certain risks and side effects. In contrast, EA offers a more convenient and safer alternative. Previous studies have shown that EA at the Yintang (GV29) and Baihui (GV20) points, as well as distal points on the heads of depressed model rats, can reverse the abnormal expression of BDNF and TrkB in the hippocampus and regulate the CaMKII signalling pathway, thereby alleviating depression‐like behaviours.PEVuZE5vdGU [15, 16, 17] Zusanli (ST36) is a crucial acupoint located on the lower limb. Previous studies have shown that stimulating ST36 effectively alleviates pain and inflammation [18, 19, 20]. In addition, studies indicate that EA at ST36 can protect against depression‐induced astrocyte atrophy in the prefrontal cortex [21]. The hippocampal functional region, particularly the CA1 area, is a key area affected in depression. However, whether EA can affect the neuronal structure and plasticity of the hippocampal functional region remains unclear.

In this study, we used a corticosterone (CORT)‐induced depression mouse model and stimulated the ST36 acupoint by EA to assess its effect on depression‐like behaviour of mice and neuronal plasticity in the hippocampal CA1 region. We aim to investigate how EA at ST36 regulates neuronal plasticity in the hippocampus and its potential role in the treatment of depression.

## 2 Materials and Methods

### 2.1 Animals

Wild type female C57BL/6 mice (aged 5–7 weeks, weighting 16–18 g) were purchased from Shanghai JieSiJie Laboratory Animal Co. Ltd. Mice were housed under controlled conditions (temperature, 24°C ± 2°C; humidity, 50% ± 10%; 12‐h light/12‐h dark cycle) with free access to food and water. The experiment began after 1‐week acclimation period. The use and care of animals complied with the guidelines of the Ethics Committee of Shanghai university, following the ARRIVE Guidelines Checklist (S1 ARRIVE Guidelines Checklist).

### 2.2 Mouse Depression Model Induced by CORT

Thirty mice were randomly assigned into three groups: the control group (vehicle), the depression model group (CORT) and the EA treatment group (EA). The mouse depression model was established using the method described in [22]. CORT (HY‐B1618, Med Chem Express) was dissolved in 0.45% β‐hydroxypropyl‐cyclodextrin (H108813, Aladdin) aqueous solution, resulting in a CORT concentration of 35 μg/mL. Mice in the depression model group and the EA treatment group were given CORT solution. Mice in the control group received 0.45% β‐hydroxypropyl‐cyclodextrin aqueous solution. Drinking water was changed every 3 days to ensure the effectiveness of CORT.

### 2.3 EA Stimulation

Mice in EA group were stimulated by EA at the ST36 on both hind limbs. ST36 is located about 4 mm away from the knee joint and 2 mm away from the anterior tubercle of the tibia, near the common fibular and tibial branches of the sciatic nerve [20]. The acupuncture needles (Hwato, ∅ 0.19 mm ×10 mm) were inserted 3 mm deep into the acupoint and connected to the EA instrument (Hwato, SDZ‐V, Suzhou Medical Instruments Co Ltd., Suzhou, China). Based on previous studies [23, 24], we selected the sparse‐dense wave type (frequency ratio 1:5) to stimulate ST36 acupoints with an intensity of 1 mA and a frequency of 2/10 Hz(2 Hz for 5 s and 10 Hz for 9 s shifting automatically) for 3 weeks (15 min/day, 6 days/week). Behavioural tests were performed at the end of the experiment.

### 2.4 Behavioural Tests

#### 2.4.1 Tail Suspension Test

Desperation behaviour in mice was evaluated by the tail suspension test (TST) according to the methods reported in [25]. The mouse's tail was taped 1 cm away from the tip, and the mouse was suspended at a height of 40 cm. The total immobility time of the mice was recorded within 5 min. The immobility time was recorded only when the mice remained completely motionless. Any instances of wriggling or climbing along their tails were excluded from the measurement.

#### 2.4.2 Forced Swimming Test

Desperation behaviour in mice was further evaluated by forced swimming test (FST) according to the method described in [26]. Mice were placed in a clear glass cylinder (height, 30 cm; diameter, 20 cm) filled with water (depth, 20 cm; temperature, 36°C ± 2°C). The tail of the mouse does not touch the bottom of the cylinder, and it cannot touch the upper edge. The total immobility time of the mice was recorded within 5 min after entering the water. Immobility is determined when an animal is floating and stops struggling, with only small limb movements to keep its head above water. Any instances of struggling or swimming were excluded.

#### 2.4.3 Open Field Test

The autonomous activity ability of mice was examined by open field test (OFT). Mice were placed in cub box (XR‐XZ301, Shanghai Soft Maze Information Technology Co. Ltd.). The mice moved in a square area (50 cm ×50 cm ×50 cm) to assess the behaviours of anxiety and motor ability. The environment is kept dark so that there is only a single constant and uniform light source in the field. Before testing, each mouse was entered into the test area for 5 min to acclimatise to the environment. The mice were placed in the centre of the open field, and the spontaneous activities of the mice were recorded with an infrared camera for 10 min. After each test, the area was cleaned to remove any residues. The behaviour trajectory, movement distance, and residence time in each area of mice were recorded by Super Maze animal behaviour analysis software.

### 2.5 Sucrose Preference Test

Reward‐seeking behaviour was assessed by sucrose preference test (SPT). According to the literature, mice were subjected to adaptive training for 48 h before the sugar preference test [27]. During the first 24 h, mice in each group were given two bottles of 1% sucrose water to get used to drinking sucrose water. In the second 24 h, one bottle of sucrose water was replaced with drinking water, and the other bottle was still 1% sucrose water, allowing the mice to become accustomed to both sucrose and pure water simultaneously. Before the test began, the mice were forbidden to eat and drink for 12 h at night. During the test, each group was given a bottle of drinking water and a bottle of 1% sucrose solution at the same time for 24 h. The bottle positions were switched at 12 h to avoid a side bias. The volume of liquid consumed from each bottle was recorded for each group. The sucrose preference index was calculated using the formula: Sucrose preference index = sucrose water consumption/total liquid consumption × 100%.

### 2.6 Golgi Staining

On the day before brain sample collection, Golgi staining solution was prepared according to the instruction of the Golgi‐Cox Kit (HTKNS1125NH, Hitobiotec Corp.), left at 20°C overnight, and the supernatant was taken for further use. The brains were perfused with normal saline before removal. A perfusion needle was inserted into the apical left ventricle of the mouse heart, and the right atrial appendage was cut open and perfused with 20 mL normal saline. After the brains were removed, they were placed into the prepared staining solution. After 24 h, the solution was renewed and soaking continued. On the 10th day, the solution was replaced by a 30% sucrose solution for dehydration. When the tissue was completely settled, the brain tissue was removed and sectioned using a freezing microtome (Leica CM 1950, Leica Biosystems). Tissue sections were 100‐μm thick and coronal. They were allowed to dry at room temperature before staining and then stained according to the instructions. Sealing was performed with resin sealant.

### 2.7 Western Blotting Analysis

After the mice were sacrificed, the brain was immediately removed and frozen at −80°C. hippocampal tissue was isolated and soaked in RIPA solution (P0038, Beyotime) supplemented with Complete protease inhibitor cocktail (04693116001, Merck) and 1 mM PMSF (ST506, Beyotime). After soaking for 2 h, the tissue was homogenised using ultrasonic apparatus. The mixture was then centrifuged at 12,000 rpm for 15 min at 4°C to extract the supernatant. Protein content was quantified using a BCA protein assay (71285‐M, Merck). The protein concentrations were adjusted to be approximately equal, and 5X SDS loading buffer (P0015, Beyotime) was added. The samples were then heated in a water bath at 95°C for 10 min to prepare them for electrophoresis.

SDS‐PAGE gels were prepared using a gel preparation kit (P0012AC, Beyotime), consisting of a 5% concentrating gel and a 10% separating gel. Samples containing 15–20 μg of protein were loaded into each well. Electrophoresis was performed at a constant voltage of 80 V until the proteins exited the concentrating gel, followed by electrophoresis at 120 V until the target proteins reached the middle of the separating gel. Proteins were then transferred to a 0.45 μm membrane using constant current electrophoresis at 200 mA. The membrane was incubated with blocking buffer (P0023B, Beyotime) in a shaking incubator for 1 h at room temperature. BDNF antibody (1:1000, ab203573, Abcam), CaMKIIβ antibody (1:1000, ab34703, Abcam), Synaptophysin antibody (1:20000, ab32127, Abcam), and GAPDH antibody (1:1000, S2118, CST) were diluted with primary antibody dilution buffer (P0023A, Beyotime) and incubated with the membrane at 4°C overnight. The following day, HRP‐conjugated secondary antibodies (1:10,000, P0216, P0208, Beyotime) were prepared and incubated with the membrane in a shaking incubator for 1 h at room temperature. The targeted protein bands were revealed using HRP substrate solution (WBKLS, Merck). After incubation, the membrane was washed three times with PBS, each for 10 min.

### 2.8 Immunofluorescence Staining

Brain tissue was perfused, fixed, dehydrated and then sectioned to a thickness of 50 μM. The sections were left at room temperature before staining and then dipped in PBS after temperature recovery. After blocking with 6% BSA containing 0.25% Triton for 1 h, antibody Ms. c‐Fos (1:1000, ab208942, Abcam) and Rb BDNF (1:1000, ab108319, Abcam), or antibody Ms. Bassoon (1:1000, ab82958, Abcam) and Rb Homer (1:1000, 160003, Synaptic Systems) were prepared with 1% BSA and incubated overnight at 4°C. Upon completion, dilutions of 594 fluorescent mouse antibody (1:1000, ab150116, Abcam) and 488 fluorescent rabbit antibody (1:1000, ab150077, Abcam) were prepared in PBS and incubated for 1 h. Finally, DAPI solution (1:1000, C1002, Beyotime) was prepared in PBS and incubated for 15 min. After the completion of each antibody incubation, the slices were washed three times with PBS for 5 min each time. When all was done, the slices were sealed with PVP sealant (C0185, Beyotime). The images were observed under a fluorescence microscope (Carl Zeiss).

### 2.9 Statistical Analysis

All statistical analyses were performed using GraphPad Prism software version 8.3.

Quantitative data were expressed as mean ± SEM, and one‐way analysis of variance (ANOVA) was used for comparison among groups. Significance was determined at the 95% confidence level. *p* < 0.05 was considered a statistically significant difference between groups.

## 3 Results

### 3.1 Inhibitory Effect of EA on Depression‐Like Behaviour Induced by CORT in Mice

**FIGURE 1:**
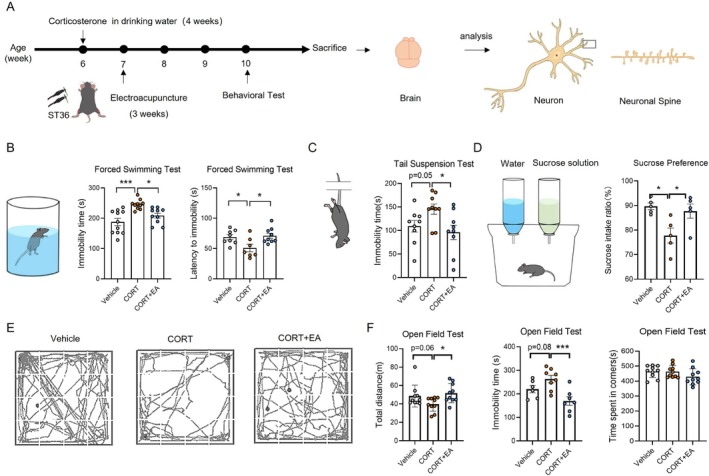
Inhibiting effect of EA stimulation at ST36 on the development of depression in mice. (A) Schematic diagram of preparation of depression model, EA treatment, behavioural tests and analysis of brain neuron. (B) Analysis of immobility time and latency period in forced swimming test. (C) Analysis of Immobility time in tail suspension test. (D) The sucrose preference index of mice in sucrose preference test. (E) The activity trace of mice in the open field test. (F) The analysis of total distance travelled, the total immobility time, and the total time spent in the corner area during open field test. *n* = 8–10. Differences among groups were evaluated by one‐way analysis of variance. *p < 0.05; **p < 0.01; ***p < 0.001.

In the state of chronic stress, the body's levels of corticosteroids are typically elevated. In this study, we employed a method described in the literature to induce a depression model using CORT in drinking water [28]. First, we observed the effects of EA on the development of depression‐like behaviour induced by CORT. The mice received CORT orally through drinking water. One week later, the mice in the treatment group were stimulated by EA at ST36 on their hind limbs once a day, 6 days a week for 3 weeks. The mice continued to drink CORT‐containing water during EA stimulation (Figure 1A). TST and FST are classical methods for assessing antidepressant‐like behaviour in rodents [29, 30]. In these tests, rodents subjected to inescapable stress conditions will maintain an immobile posture. Antidepressant treatments are known to reduce the duration of immobility. Therefore, at the end of the experiment, behavioural tests such as TST, FST and OFT were used to evaluate the degree of depression in mice. Compared with normal mice, the immobility time of mice in the CORT group in the FST (Figure 1B) and the TST (Figure 1C) significantly increased, along with a decreased latency to immobility in FST, indicating the presence of desperate behaviour. In addition, mice in the CORT group showed a reduced preference for sugar water (Figure 1D), reflecting diminished reward‐seeking behaviour and a decreased ability to experience pleasure. In contrast, the immobility time of mice in the EA group showed a significant reduction in immobility time during both the TST and FST, along with an increased latency to immobility (Figure 1B,C). Furthermore, the consumption of sugar water was significantly increased compared to the model group (Figure 1D). These findings suggest that EA effectively inhibits the development of desperate behaviour and the loss of reward‐seeking behaviour induced by oral CORT.

We also assessed the autonomous activity and exploration behaviour of mice in an open field. As shown in Figure 1E,F, the activities of mice in the CORT group in the open field were significantly reduced, evidenced by a marked decrease in total distance travelled and a significant increase in immobility time. This indicates a decline in the autonomous ability of the mice in the CORT group. In contrast, the mice in the EA group demonstrated an increase in total distance travelled in the open field and a significant reduction in immobility time compared to the CORT group (Figure 1E,F), suggesting that EA effectively inhibited the decline of autonomous behaviour induced by oral CORT. These behavioural results suggest that EA stimulation at ST36 can significantly alleviate depression‐like behaviour induced by CORT and inhibit the development of depression.

**FIGURE 2:**
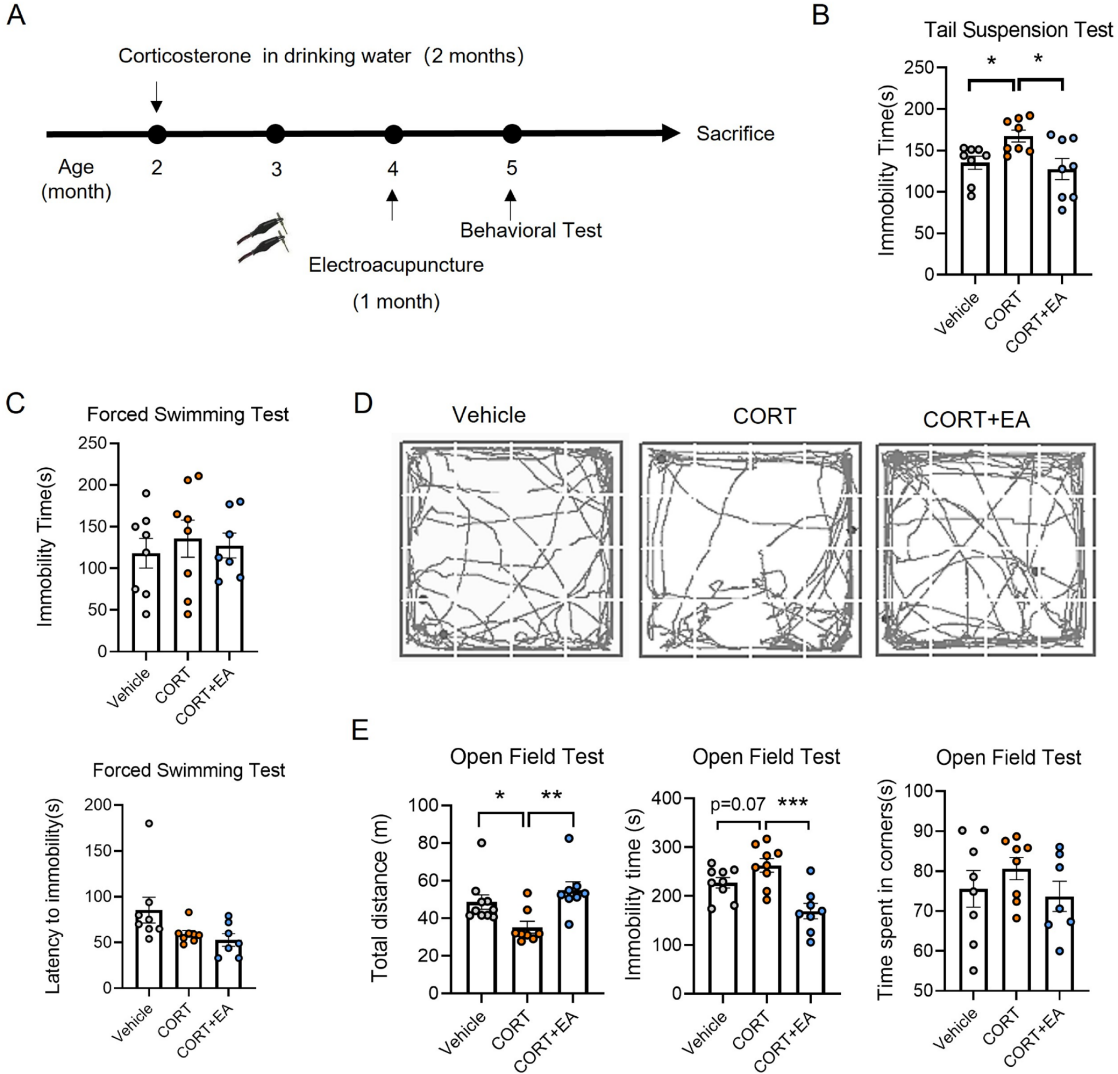
Therapeutic effect of EA stimulation at ST36 on depression model mice. (A) Schematic diagram of preparation of depression model, EA treatment and behavioural tests. (B) Analysis of immobility time in tail suspension test. (C) Analysis of immobility time and latency period in forced swimming test. (D) The activity trace of mice in open field test. (E) The analysis of total distance travelled, the total immobility time and the total time spent in the corner area in open field test. *n* = 8. Differences among groups were evaluated by one‐way analysis of variance. *p < 0.05; **p < 0.01; ***p < 0.001.

To investigate the therapeutic effect of EA on a mouse model of chronic depression, we established the model using a standardised method. First, the mice were given CORT‐laced drinking water for 2 months. After withdrawal of CORT, the mice in the treatment group received EA stimulation at ST36 acupoint for 1 month (Figure 2A). As shown in Figure 2, mice in the CORT group showed a significant increase in immobility time during the TST compared with normal mice (Figure 2B), as well as an increase in immobility time and a decrease in latency during the FST (Figure 2C). These findings suggest that depression‐like behaviour induced by long‐term CORT intake does not spontaneously recover after discontinuation of administration. Compared with mice in the CORT group, mice in the EA group showed a significant reduction in immobility time during the TST (Figure 2B), while the latency in FST was not significantly affected, although a trend toward decreased immobility time was observed (Figure 2C). This indicates that EA alleviates the desperate behaviour induced by long‐term CORT exposure in mice. Results from the OFT revealed that the CORT group exhibited significantly reduced activity levels and total distance travelled, as well as increased immobility time and a tendency to stay in corners compared with the untreated group (Figure 2D,E). In contrast, the EA group showed a significant increase in total distance travelled and a significant decrease in immobility time compared with the CORT group (Figure 1D,E). This showed that after EA treatment, the ability to move autonomously was also restored in mice that drank CORT for a long time. These results indicate that EA treatment can significantly alleviate desperate behaviours in mice with depressive symptoms and restore their ability to engage in autonomous movement.

### 3.2 Effects of EA on Neuronal Structure in the Hippocampal CA1 Region in a Mouse Model of Depression

**FIGURE 3:**
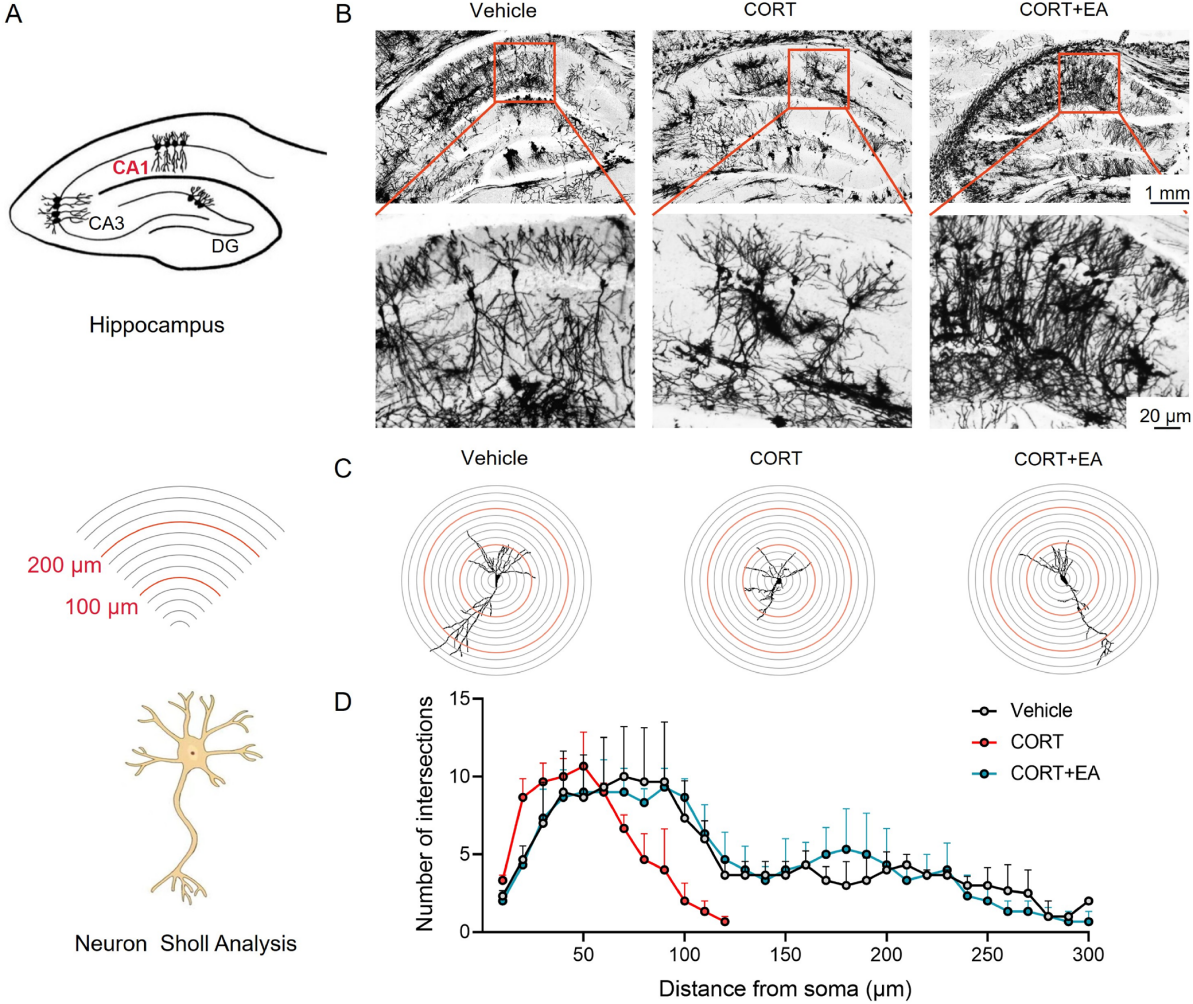
Protective effect of EA stimulation at ST36 on neurons in hippocampal CA1 region. (A) Schematic diagram of functional structural partitions of hippocampus and Sholl analysis of neurons. (B) Image of Golgi‐cox staining in the hippocampus of mice, with a scale bar of 1 mm for the overall image and 20 μm for the CA1 region. (C) Sholl analysis of neurons in CA1 region. (D) Statistical graph of Sholl analysis of neurons in CA1 region. *n* = 6.

Based on the observation that EA alleviated depression‐related symptoms, we used Golgi staining to further investigate neurons in the hippocampus of the mouse brain, focusing on the changes in the number and structure in the CA1 region (Figure 3A). As shown in Figure 3, the number of neurons in the hippocampus of mice in the CORT group was significantly lower than that of the normal group, with a sparse distribution of neurons (Figure 3B). This finding indicates that depression leads to a decrease in hippocampal neurons in mice. Sholl analysis results showed a significant decrease in the complexity of neuronal dendrites in the hippocampal CA1 region of the CORT group, characterised by reduced dendritic length and fewer intersections with other neurons (Figure 3C,D). In contrast, the distribution of neurons in the hippocampal CA1 region of the EA group was normal, exhibiting a dense population of neurons with intact structure (Figure 3B). The length of dendrites and the number of branches remained comparable to those in the normal group (Figure 3C,D). These findings suggest that EA therapy effectively attenuated CORT‐induced reduction and structural damage in the hippocampal CA1 region.

### 3.3 EA Regulates Neuronal Plasticity by Upregulating the Levels of BDNF and CaMKIIβ Proteins

**FIGURE 4:**
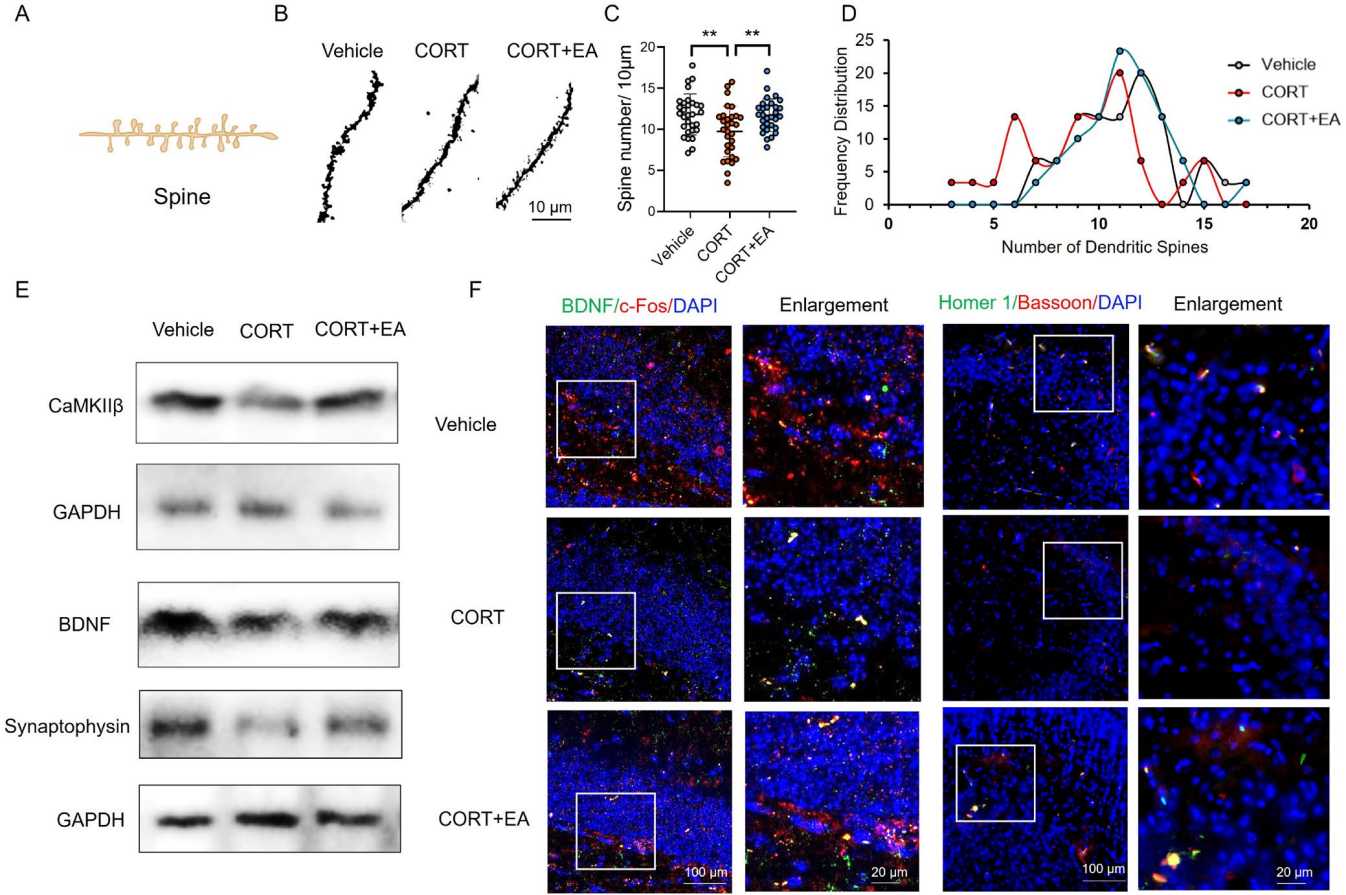
Analysis of neuronal plasticity and related regulatory proteins in the hippocampus. (A) Schematic diagram of dendritic spines in hippocampus. (B) Image of dendritic spines of neurons in CA1 region of hippocampus (scale bar = 10 μm). (C) Statistical graph of the dendritic spine number of neurons in CA1 region. *n* = 30. Differences among groups were evaluated by one‐way analysis of variance. *p < 0.05; **p < 0.01; ***p < 0.001. (D) Distribution trend of dendritic spine numbers of neurons in CA1 region. (E) Expression levels of synaptophysin, BDNF, and CaMKIIβ proteins in mouse hippocampal tissues were analysed by Western blotting. (F) Immunofluorescence co‐localization staining images of c‐Fos and BDNF proteins, the presynaptic membrane bassoon protein and postsynaptic membrane homer 1 protein in the hippocampus (scale bar = 100 μm; scale bar of enlarged image = 20 μm).

We further analysed the number of dendritic spines in hippocampal neurons, which directly reflects neuronal plasticity changes (Figure 4A). Compared with the normal group, there were marked morphological changes in dendritic spines in the hippocampus, accompanied by a significant reduction in number (Figure 4B–D). These results suggest that CORT exposure causes substantial impairment of neuronal integrity and plasticity in the hippocampal CA1 region, which is a characteristic pathological change associated with depression. In the EA group, the number of dendritic spines on neurons was similar to that of the normal group without significant reduction (Figure 4B–D).

**FIGURE 5:**
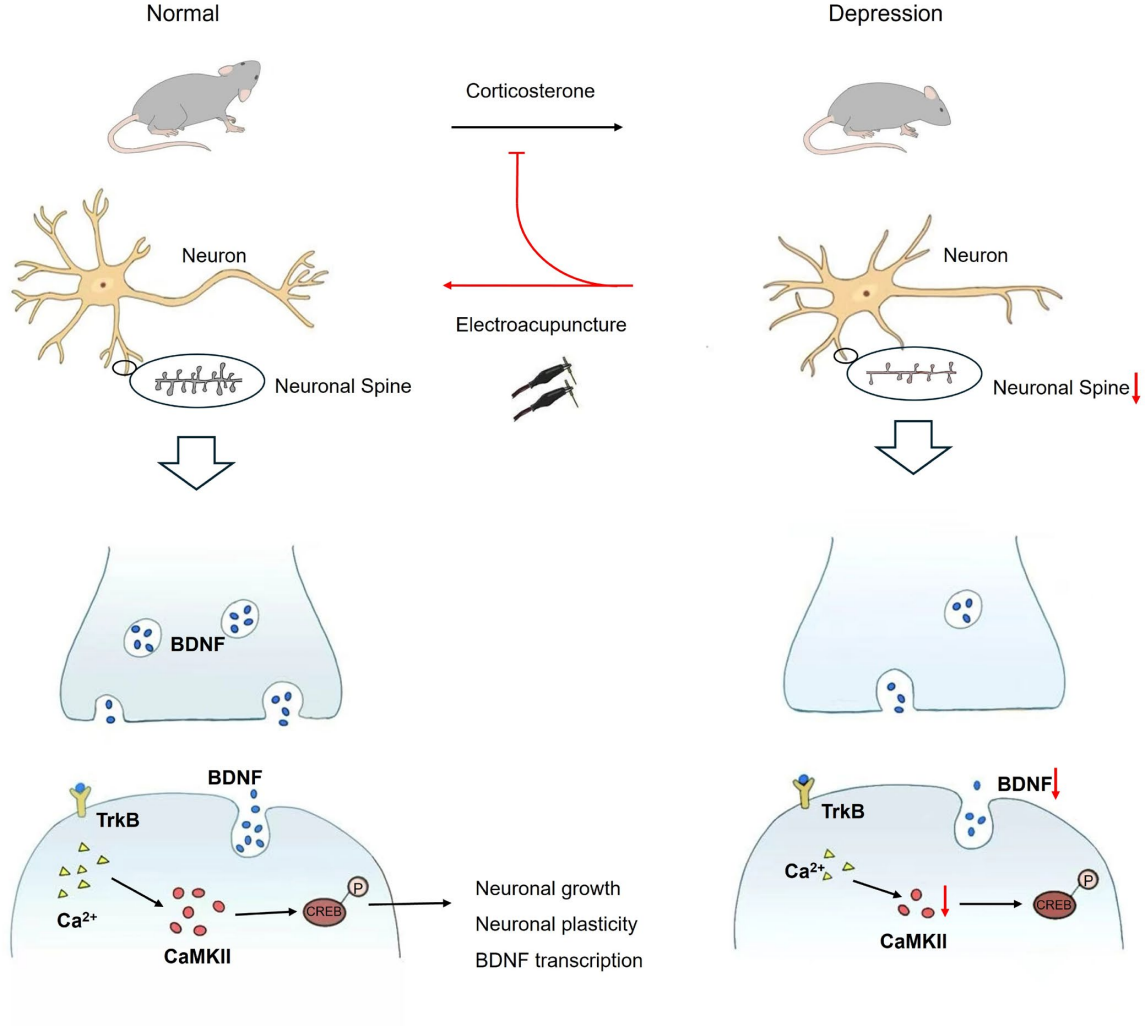
Schematic summary of electroacupuncture regulating neuronal plasticity in depression model mouse. Corticosterone‐induced depression‐like symptoms in mice, with decreased neuronal plasticity in the hippocampus. EA effectively prevents and alleviates the symptoms of depression in the corticosterone‐induced mouse model. It regulates the expression of BDNF and protects the neuronal integrity and plasticity in the hippocampus by regulating the TrkB/CREB/CaMKIIβ signalling pathway.

To elucidate the underlying mechanism of EA in protecting neuronal plasticity, we further analysed the expression of synaptophysin, BDNF and CaMKIIβ proteins in hippocampus, which are closely related to neuronal plasticity. The levels of these proteins in the hippocampus were assessed using Western blot analysis. In the context of hippocampal neuronal plasticity, BDNF activates downstream signalling pathways leading to CaMKIIβ phosphorylation, which in turn activates CREB phosphorylation to promote neuronal growth and protect neuronal plasticity. The results showed that expression levels of synaptophysin, BDNF, and CaMKIIβ proteins were significantly reduced in the hippocampus of the CORT group compared with the normal group (Figure 4E). Immunofluorescence staining further confirmed that the expression levels of BDNF and c‐Fos in the hippocampus were decreased in the CORT group (Figure 4F). Simultaneously, there was a significant decrease in the expression of the presynaptic membrane bassoon protein and postsynaptic membrane homer 1 protein. The activity of hippocampal neurons in the CORT group was inhibited, resulting in the downregulating of BDNF and CaMKIIβ signalling pathways and the reduction of related protein expression. However, there was no significant decrease in the expression of synaptophysin, BDNF and CaMKIIβ proteins in hippocampal tissue of mice in the EA treatment group (Figure 4E), and neuronal activity remained relatively unchanged (Figure 4F). This suggests that EA therapy effectively activates neurons in the hippocampus, preventing CORT‐induced decline in BDNF levels and inhibition of associated regulatory pathways. EA stimulation at acupoints helps maintain the expression of BDNF and CaMKII proteins by activating neurons, thereby protecting the integrity of neuronal structure in the hippocampus and preserving the plasticity of dendritic spines (Figure 5).

## 4 Discussion

In recent years, the incidence of depression has been steadily increasing, with a notable trend toward younger people. With the outbreak of the COVID‐19 pandemic over the past few years, the situation is accelerating [31]. Therefore, the early and effective treatment of depression has received increasing attention. Currently, the commonly used clinical treatment methods mainly include psychological counselling and pharmacotherapy. Psychological counselling requires long treatment cycles and is expensive. Pharmacotherapy, although effective, requires consideration of drug absorption across the blood–brain barrier and may be associated with systemic side effects. Acupuncture, as an effective and safe form of physical therapy, shows good application potential in this context. Our study found that EA can regulate the plasticity of neurons in the hippocampus, which is closely related to depression. This further proves the superiority of acupuncture in treating depression and provides valuable insights into the mechanisms underlying its therapeutic effects.

Chronic mental tension and stress can lead to depression, often accompanied by elevated levels of adrenocortical steroids. In animal studies, chronic stress stimuli or CORT administration is often used to establish models of depression. CORT is an adrenocortical hormone that corresponds to cortisol produced by humans in response to stress. Prolonged high levels of CORT can induce depression, making it a widely used agent for constructing depressed mouse models, usually administered by subcutaneous injection or by drinking water. In this study, we used CORT to prepare drinking water to establish a depressed mouse model and subjected the mouse model to EA stimulation at the ST36 acupoint. We aimed to verify the preventive and therapeutic effects of EA on depression‐like behaviour, as well as the protective effect on neurons in the hippocampus. Our study found that EA therapy was effective against CORT‐induced depression‐related development of desperate behaviour, as well as deficits of reward behaviour and autonomic actions. Furthermore, for mice that have already developed depression, EA stimulation also has a significant therapeutic effect. These results suggest that EA holds promise as a viable treatment option for depression.

The hippocampus is one of the key brain regions in response to depression. Hippocampal volume atrophy is the most typical symptom of depression and serves as one of the evaluation criteria for major depressive disorder [4, 32]. Its severity is directly related to the duration and recurrence of depression. In a CORT‐induced depression model, there is significant volume shrinkage in the CA1 region of the hippocampus, decreased neuronal excitation, and reduced neuronal connection [6, 30]. Neuronal plasticity is the ability of neurons to make new connections, and neuronal plasticity in the hippocampus is associated with learning, memory and emotion regulation [33, 34]. Impairment of hippocampal function can lead to depression, while restoration of hippocampal damage can alleviate depression‐related symptoms [35, 36]. In this study, we performed Golgi staining of brain tissue, Sholl analysis of neurons, and statistical distribution of neuronal dendritic spines to assess neuronal plasticity in the CA1 region of the hippocampus. The results showed that EA treatment prevented depression‐related neuronal damage in the hippocampal CA1 region and preserved neuronal integrity and plasticity. This preservation is crucial for maintaining the function of the hippocampus. This suggests that EA stimulation may alleviate depression‐like behaviour through a regulatory pathway related to neuronal plasticity.

We further investigated the TrkB/CREB/CaMKIIβ regulatory pathway, which plays a key role in depression. BDNF protein, an upstream signalling molecule that activates this pathway, was also detected. Previous studies have shown that BDNF is involved in the regulation of neuronal plasticity and the activation of neurons in the hippocampus [37]. Clinically, a variety of antidepressant drugs target the BDNF pathway to induce downstream signalling pathways by activating the BDNF receptor TrkB, thereby achieving antidepressant effects [38]. Our study found that EA treatment effectively reversed the depression‐associated inhibition of neuronal activity, upregulated the expression levels of BDNF protein, and maintained expression levels of CaMKIIβ protein(Figure 5). This suggests that EA enhances BDNF expression, thereby activating the TrkB/CREB/CaMKIIβ regulatory pathway, which may play an important role in the treatment of depression‐like behaviour.

## 5 Conclusion

In conclusion, this study demonstrates that EA stimulation of ST36 acupoint has a beneficial effect on the prevention and treatment of depression. EA at ST36 can regulate the expression of BDNF and protect the neuronal integrity and plasticity of the hippocampus via regulating the TrkB/CREB/CaMKIIβ signalling pathway. As a non‐invasive form of physical stimulation, EA has the potential to serve as a safer treatment for depression. Our findings shed light on the underlying mechanism by which EA alleviates depression to some extent, providing an experimental basis for its clinical application. In the future, the in‐depth mechanism of EA stimulation improving neuronal plasticity needs to be further explored.

## Author Contributions

Y.S. and L.W. designed the study. Y.W., X.D., C.D., and M.W. performed most of the experiments. Y.W., C.D., and Y.S. performed the data analysis for the study. L.W., Y.Z. and J.H. supervised the study. Y.W. and Y.S. wrote the paper.

## Conflicts of Interest

The authors declare no conflicts of interest.

## Data Availability

The data that support the findings of this study are available from the corresponding author upon reasonable request.
